# Metabolic aspects of surgical subcutaneous fat removal: An umbrella review and implications for future research

**DOI:** 10.17305/bjbms.2022.8175

**Published:** 2023-03-16

**Authors:** Saif Badran, Suhail A Doi, Moustapha Hamdi, Atalla Hammouda, Sara Alharami, Justin Clark, Omran A H Musa, Abdul-Badi Abou-Samra, Abdella M Habib

**Affiliations:** 1 Department of Population Medicine, College of Medicine, QU Health, Qatar University, Doha, Qatar; 2 Department of Plastic and Reconstructive Surgery, Brussels University Hospital, Vrije Universiteit Brussel (VUB), Brussels, Belgium; 3 Department of Plastic Surgery, Hamad General Hospital, Doha, Qatar; 4 Institute for Evidence-Based Healthcare, Faculty of Health Sciences & Medicine, Bond University, Gold Coast, Queensland, Australia; 5 Department of Medicine, Weill Cornell Medicine Qatar, Qatar Foundation, Doha, Qatar; 6 Qatar Metabolic Institute, Hamad Medical Corporation, Doha, Qatar; 7 College of Medicine, QU Health, Qatar University, Doha, Qatar

**Keywords:** Surgical subcutaneous fat removal (SSFR), body contouring surgery, metabolism, insulin resistance, inflammation, adipokines

## Abstract

Although obesity is a preventable disease, maintaining a normal body weight can be very challenging and difficult, which has led to a significant increase in the demand for surgical subcutaneous fat removal (SSFR) to improve physical appearance. The need for SSFR is further exacerbated because of the global rise in the number of bariatric surgeries, which is currently the single most durable intervention for mitigating obesity. Fat tissue is now recognized as a vital endocrine organ that produces several bioactive proteins. Thus, SSFR-mediated weight (fat) loss can potentially have significant metabolic effects; however, currently, there is no consensus on this issue. This review focuses on the metabolic sequelae after SSFR interventions for dealing with cosmetic body appearance. Data were extracted from existing systematic reviews and the diversity of possible metabolic changes after SSFR are reported along with gaps in the knowledge and future directions for research and practice. We conclude that there is a potential for metabolic sequelae after SSFR interventions and their clinical implications for the safety of the procedures as well as for our understanding of subcutaneous adipose tissue biology and insulin resistance are discussed.

## Introduction

Obesity has reached pandemic levels and currently affects all age groups and socioeconomic classes worldwide. Obesity prevalence has almost tripled in the last 50 years according to the World Health Organization and this, in turn, has led to more fatality than malnutrition and being underweight combined [1]. The rising obesity rate has led to a substantial rise in metabolic diseases, such as diabetes mellitus type 2 (T2D), hypertension, cardiovascular disease, non-alcoholic hepato-steatosis, and dyslipidemia [2].

Lipids comprise a wide range of molecules, such as phospholipids, fatty acids, and triglycerides [3]. These molecules represent a highly efficient energy resource. Recent studies have advanced our view of adipose tissue from being simply an energy store, into an active endocrine organ, which secretes several metabolically active adipokines, such as leptin, adiponectin, and resistin. The latter plays an essential role in glucose hemostasis and energy metabolism in our body [2]. These molecules have been ascribed to have a critical role in energy homeostasis through communication with organs that maintain system-wide metabolic homeostasis such as the liver. Of the adipocyte-derived factors, adiponectin and leptin are among the essential adipokines. Indeed, adiponectin analogs are now considered one of the promising new therapeutic targets for obesity-linked hyperglycemia, that mitigates obesity and improves insulin sensitivity [4].

Insulin resistance, as a consequence of such dysregulation associated with obesity, is what links the latter to T2D. Insulin resistance leads to dysregulation of glucose homeostasis via a combination of impaired glucose clearance and elevated glucose production in the liver. Adipose tissue is a major contributor to insulin sensitivity/resistance status. Too little fat mass, as seen in patients with lipodystrophy, results in a severe form of insulin resistance, and too much adipose mass can also result in a similar condition [5]. The primary reason for the latter form of insulin resistance may be hypoxia in adipose tissue that leads to inflammatory lipo-toxicity [6].

Currently, it is unknown if the removal of excess subcutaneous fat tissue through surgical subcutaneous fat removal (SSFR; also known as body contouring surgeries such as liposuction or abdominoplasty) ameliorates the mass of hypoxic fat thus reducing its consequences. Such surgeries have become very common because, although obesity can be prevented, maintaining a normal body weight can be very challenging and difficult and the increase in demand for SSFR has been driven by patients seeking an improved physical appearance [7]. However, the precise effect of sudden removal of a patient’s body fat on metabolism is still not fully understood.

### Surgical subcutaneous fat removal

The current drift toward cosmetic plastic surgeries, especially the body contouring surgeries which aim to produce a more attractive body shape by removing the excess of skin and fat tissue from multiple body areas, is due to several reasons such as the increase in the safety of these procedures, the increase in the availability of these operations, and largely due to the recent increase in the number of bariatric surgeries. Bariatric surgery is performed for morbidly obese patients to facilitate loss of a significant amount of their body fat mass. Because of the rapid and massive weight loss following bariatric surgery such as sleeve gastrectomy, many patients tend to require body contouring plastic surgery to remove redundant skin and excess body fat [8]. The body-contouring surgery is also done for purely cosmetic purposes in patients not undergoing bariatric surgery.

A typical example of these body contouring surgeries is the abdominoplasty (as known as Tummy Tuck) surgery which suddenly removes around 2–3 kg of abdominal subcutaneous fat (ASF) tissue, and usually is followed by tightening of the abdominal wall muscles, to correct divarication of recti muscles [9]. The other commonly undertaken surgery is suction-assisted lipectomy and, together with abdominoplasty, these represent the commonest plastic surgery procedures that target subcutaneous fat from unwanted areas such as the abdominal wall and flanks. The accelerating demand for these surgical procedures has gradually moved the practice from removing a small amount of intractable fat tissue to the removal of a large volume (more than five liters) of subcutaneous fat tissue, which eventually can result in a significant metabolic effect [10]. However, whether the metabolic effects of these two surgeries are the same or different is not known. In fact, previous reviews and meta-analyses (MAs) have combined these two procedures together which might not be accurate. For example, the repair of the abdominal wall in abdominoplasty might result in an increased intra-abdominal pressure with reduced space for the future expansion of intra-abdominal fat tissue, which might result in different metabolic effects than liposuction [10, 11].

Finally, a distinction needs to be made between SSFR and other modalities of fat loss (such as diet, exercise, or bariatric surgeries) in that non-SSFR modalities result in a gradual decrease in both the subcutaneous and intra-abdominal fat tissue. This gradual reduction occurs through a decrease in the size of the adipocytes while with SSFR there is actual loss of subcutaneous adipocyte numbers, but without impact on intra-abdominal adipocytes.

### Fat removal sites in SSFR

SSFR classically is from abdominal and thigh areas, although other sites may less commonly be targets for surgery. Abdominal (or upper-fat) distribution is correlated more strongly with obesity-associated metabolic risks and consequences than the gluteo-femoral (or lower-fat) distribution in the gluteal and thigh regions [12]. Fat in the abdomen may be subcutaneous (ASF) or as abdominal visceral fat (AVF) tissue and it should be noted that only ASF is the target for abdominal SSFR [9]. AVF is intraperitoneal fat that represents both the mesenteric as well as the omental fat cells [13]. AVF is typically formed of large adipocytes and contains necrotic and inflammatory tissues. There is also retroperitoneal fat in humans of unclear significance.

Central obesity in the abdominal area represents one of the essential components of metabolic syndrome, along with insulin resistance, elevated serum triglyceride, blood pressure, and low high-density lipoproteins. The distribution of fat deposits in the abdomen (ASF vs AVF) has thus been thought to determine metabolic outcomes and that AVF tissue is more “pathogenic” [14] and is what has been linked to metabolic syndrome and T2D [15]. Other studies have also proposed that both ASF and AVF play a role in metabolic risk [10] but largely the metabolic risk of obesity has been linked mainly to AVF because it is directly involved in the delivery of free fatty acids as well as inflammatory proteins such as interleukin-6 (IL-6), to the liver via the portal circulation [16]. It is nevertheless probable that ASF may also play a role given that more than 80% of the free fatty acids and other inflammatory proteins reach the liver via the systemic circulation [17]. This is supported by studies that report the intrahepatic triglyceride rather than AVF is a better marker for obesity-associated metabolic risk [18]. Therefore, it has recently been suggested that the metabolic risk in obesity is a shared effect of molecules secreted by both these compartments. Thus, there is an expectation that SSFR may alter glucose homeostasis and insulin resistance as a direct consequence of surgical ASF removal.

### Potential for metabolic sequelae after SSFR

Research has found that even a small weight loss of ten percent can result in a significant improvement of obesity-linked metabolic abnormalities, such as insulin resistance, high blood pressure, and abnormal inflammatory marker levels [19, 20]. Additionally, increased knowledge of the metabolic consequences of excess body fat and observations after bariatric surgeries [21] have suggested that there could possibly be a similar effect after SSFR. This has been examined in several studies, which measure hormonal changes before and after SSFR at different time points. These studies have been small and heterogeneous and have reported inconsistent effects on metabolic parameters, such as insulin resistance, adipokine levels, and inflammation [22–34] To improve power and resolve the inconsistency, these studies have been combined in several syntheses, both systematic reviews (SRs) and MAs. The aim of this umbrella review therefore is to now examine these syntheses and summarize their findings as well as define current knowledge gaps in the metabolic impact of SSFR, particularly, changes in insulin resistance, inflammatory markers, and adipokines levels.

## Materials and methods

### Study inclusion and exclusion criteria

A search was conducted for evidence syntheses that synthesized data on the metabolic changes after SSFR. PubMed, Embase, and Scopus databases were searched without any date, language, or publication restriction but exclusion of non-English and animal studies, as well as non-surgical body fat removal and bariatric surgeries.

### Search strategy

**Figure S1.**
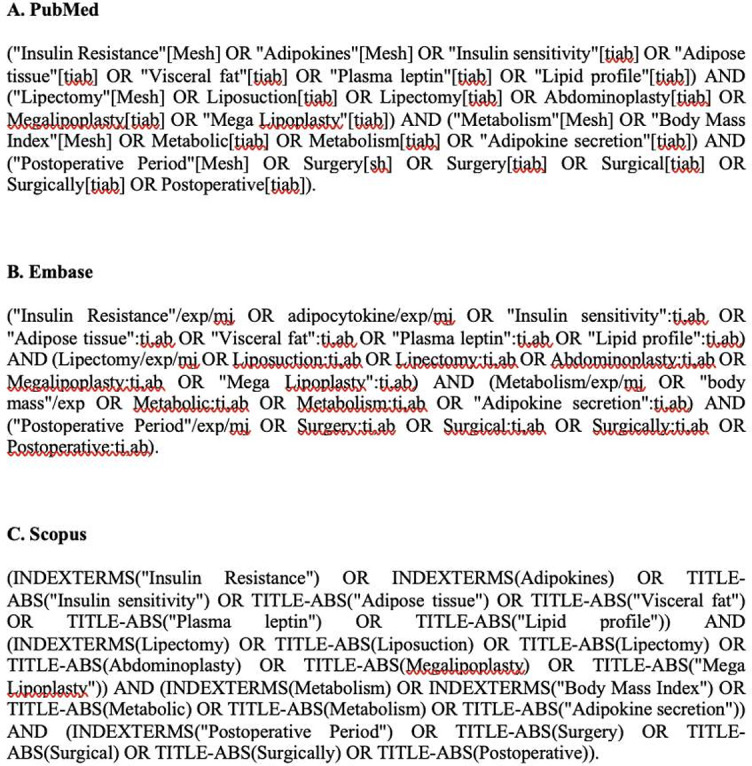
Search strings.

Search was conducted on 8 November 2021 by two independent authors using the polyglot Search Translator [35]. The search strings used are given in the supplementary material (Figure S1) for the syntheses that report changes in insulin sensitivity, inflammatory markers, and adipokines levels after SSFR. Data were extracted regarding synthesis type (SR or MA), title and author, year of publication, type of SSFR, a summary of included studies, follow-up duration after SSFR, and possible evidence gaps. Main findings were summarized regarding metabolic changes in terms of potential inflammatory and anti-inflammatory adipokines and other metabolic markers.

### Quality assessment

A MeaSurement Tool to Assess systematic Reviews-2 (AMSTAR-2) was used to assess the quality of the included reviews and each included synthesis was examined against 16 quality safeguards to assess their methodological quality [36].

### Data synthesis

A structured summary of findings was done for the eligible and included SRs and MAs. Metabolic change findings were assessed in three categories: insulin resistance, inflammatory markers, and adipokines. For each of the categories, a separate table of findings was formulated.

## Results

### Study selection

**Figure 1.**
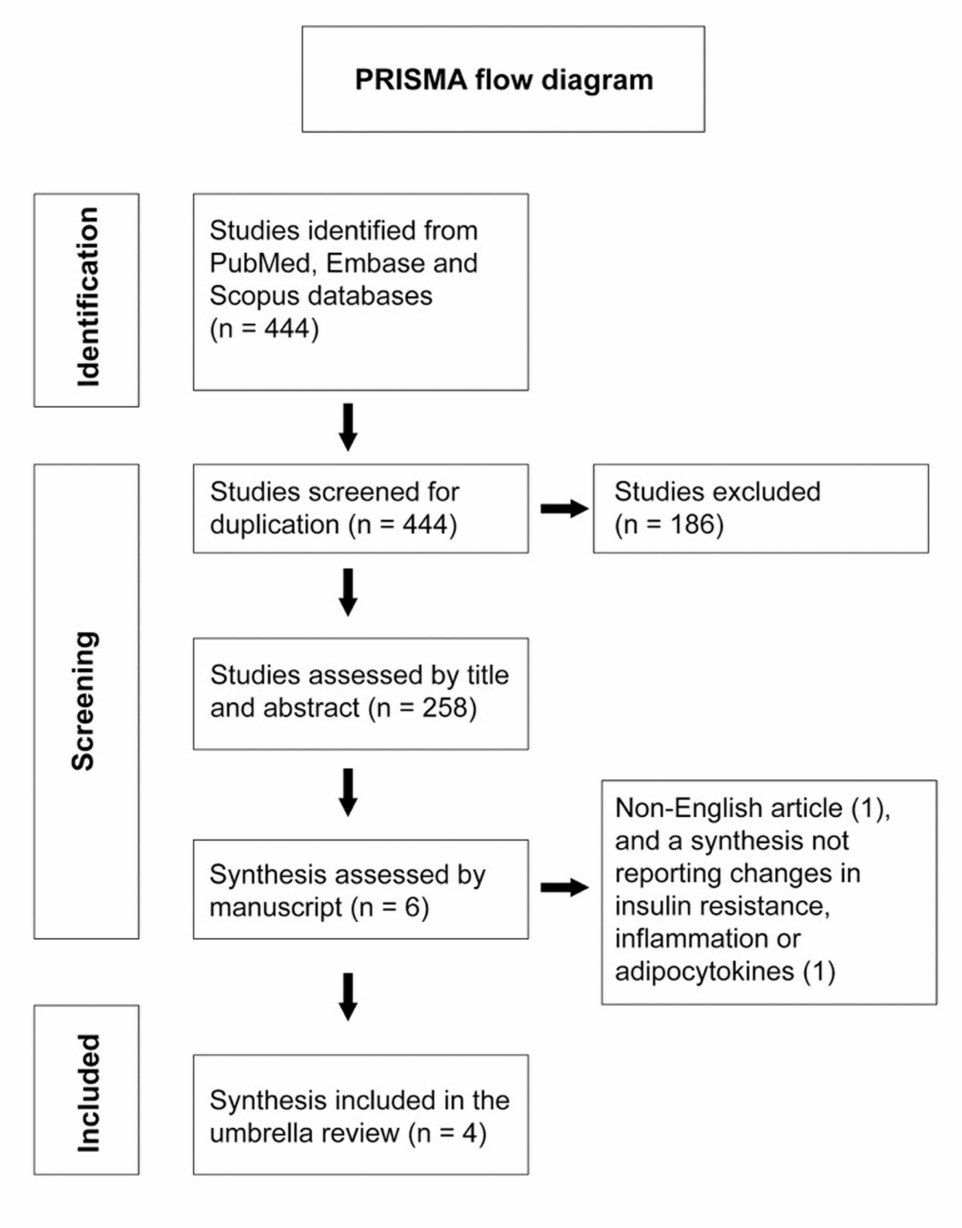
PRISMA flow diagram of the study selection process.

A search in the three databases: PubMed, Embase, and Scopus on (08/11/2021) resulted in 444 studies. A total of 186 duplicate studies were excluded. The remaining 258 articles were screened by title and manuscript for eligibility of which six met inclusion criteria. One synthesis was in French and was excluded from this umbrella review [37], while another was excluded as it reported changes in weight and fat mass only [38]. There were thus three MAs and one SR included, and Figure 1 depicts the PRISMA flow diagram for the selection of studies.

**Figure S2.**
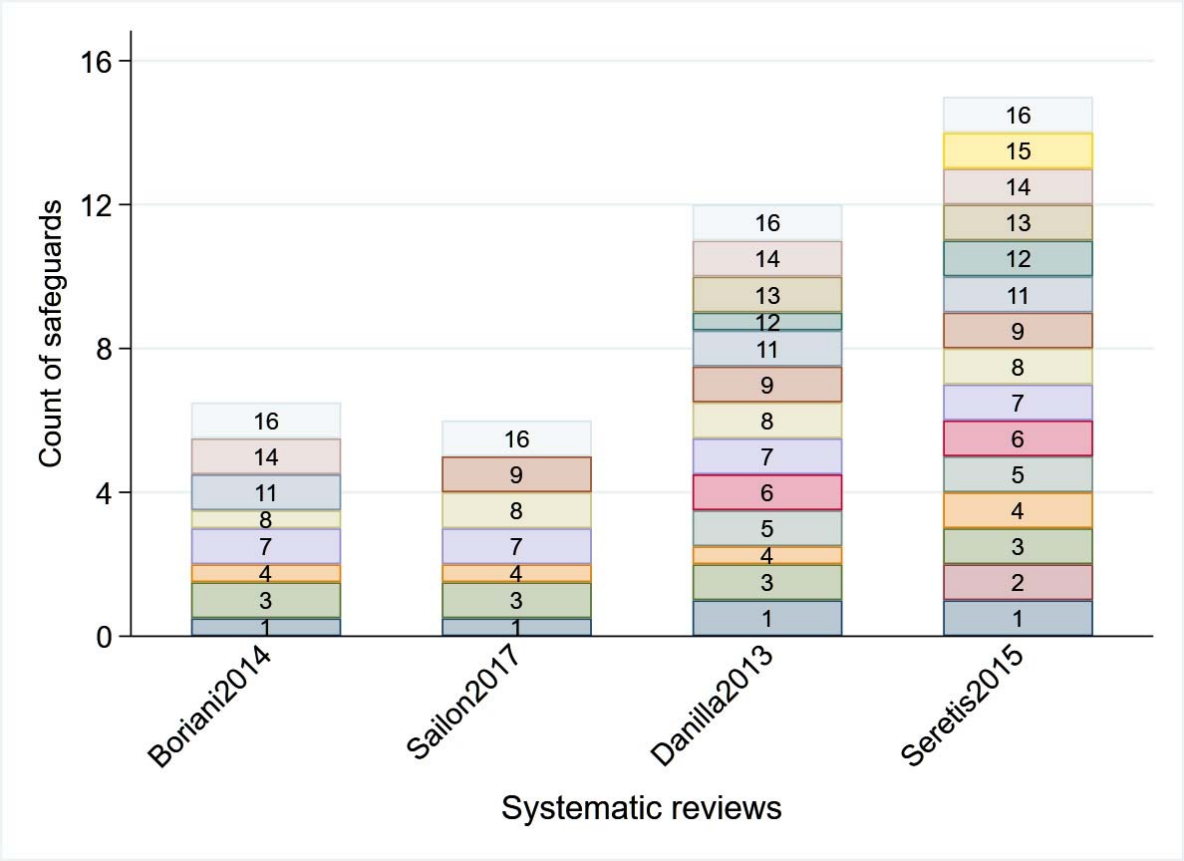
**Quality assessment of the four syntheses.** The thick boxes indicate presence of the quality safeguard and the thin boxes indicate they are partially present. Missing numbers indicate the safeguard was missing.

The first synthesis was conducted in 2013 [39], and since then, another three syntheses have been published [10, 11, 40]. None of the four included syntheses (15, 14, 12, and 11 studies included) examined the time trend after SSFR, and thus they looked at metabolic changes through quantitative analyses (if any) did not consider the heterogeneity in follow-up duration across studies. This umbrella review summarizes the changes reported in three categories: insulin resistance, inflammatory markers, and adipokines levels. Quality assessment of the included syntheses demonstrated that most of them included PICO components in the review, explanation of inclusion criteria, justification for the excluded studies, use of a satisfactory quality assessment tool in studies included in the review, and adequate description of the included studies. See supplementary material Figure S2.

### Impact of SSFR on insulin resistance

Several studies have measured changes in insulin resistance status after SSFR using different tests, such as measuring fasting glucose, fasting insulin, and the Homeostatic Model Assessment for Insulin Resistance (HOMA-IR) [31, 34, 41], insulin tolerance test (ITT) [42], oral glucose tolerance test [30, 43], and the gold standard glucose clamp test [29, 44]. Apart from the glucose clamp test, most of these tests are not accurate in assessing the change in insulin sensitivity, and the studies that used the glucose clamp test had a small sample size and a lot of variability among participants in terms of diabetic status, and degree of obesity. The challenge behind using accurate tests such as the hyper insulinemic glucose clamp and the intravenous glucose tolerance test is the fact that they are very demanding [45].

Across three MAs and one SR examining the effect of SSFR on insulin sensitivity, most of the evidence suggests a possible improvement in obesity-associated insulin resistance, however, there was a lack of clarity regarding the extent of the effect and clinical significance. This was because there were major problems in the design and analysis of the MAs and therefore results couldn’t be thus interpreted. In terms of the SRs, there was no clarity on the extent of the changes across the studies since there was a focus on statistical significance only. In summary, syntheses were inconsistent, there was a trend toward improvement in insulin sensitivity, and the clinical extent or duration of any improvement remains unclear. The impact of SSFR on insulin resistance thus remains unknown given the data reported in (Table 1) and we recommend that a dose response MA be conducted to answer this question.

**Table 1:** Syntheses that report changes in insulin sensitivity after SSFR

	**Synthesis author and year**	**Synthesis type**	**Type of SSFR**	**Included studies**	**Follow up**	**Main finding**	**Remaining evidence gaps**
1	Sailon et al. 2017 [10]	SR	Liposuction	Ten prospective studies (346 participants), which examined large volume liposuction (>3.5 liters)	3 weeks–6 months	Author reported conflicting results but stated that surgical fat removal by large volume liposuction can improve insulin sensitivity. No clear extent of change was reported.	This SR focused examining the statistical significance of these changes post SSFR, without reporting the extent of change, or its clinical importance. The review had substantial heterogeneity in terms of participants baseline characteristics, included studies sample size, and different assessment tools for insulin resistance.
2	Seretis et al. 2015 [11]	MA	Liposuction + Abdominoplasty	Four studies (140 participants)	2 months–2 years	Fasting glucose levels changes after SSFR were not statistically significant (1.42, 95% CI: −1.57, 4.40). Changes in insulin sensitivity were also assessed either by insulin tolerance test or HOMA index, however the result reported a lack of significant change after SSFR (0.14, 95% CI -0.69–0.96).	This MA included studies that were so contrived in terms of control group that no conclusion was possible. The small number of studies limited its validity and prevented subgroup analysis according to certain confounders such as age or BMI.
3	Boriani et al. 2014 [40]	MA	Liposuction	Five prospective studies (190 participants)	3 months–1 year	Fasting insulin levels were significantly higher before SSFR by a weighted mean difference of 3.49 mIU/ml (95% CI 1.12, 5.87).	There was a degree of heterogeneity among studies (*p* ═ 0.02, I^2^ ═ 67%). Fasting insulin levels were used as a surrogate for insulin resistance, which is an indirect measure.
4	Danilla et al., 2013 [39]	MA	Liposuction	Five quasi experiment studies (111 participants)	3 weeks–1 year	Analysis reported that SSFR result in decreased fasting insulin levels, and the amount of reduction was associated with the amount of aspirated fat, independent with the baseline BMI. No significant change was reported in HOMA-IR levels after SSFR.	Although this MA studied the effect of time on the SSFR induces changes in insulin resistance, the sample size of the included studies was small.

SSFR: Surgical subcutaneous fat removal; SR: Systemic review; MA: Meta-analysis; CI: Confidence interval; BMI: Body mass index; HOMA-IR: Homeostatic model assessment for insulin resistance.

### Impact of SSFR on inflammation

Obesity is associated with chronic low-grade inflammation. This is a result of the increased influx of immune cells to the fat tissue, as well as the increased secretion of inflammatory cytokines such as tumor necrosis factor-alpha (TNF-α) [2]. Adipocytes have an equal proinflammatory effect on the macrophages [46]. This inflammatory status is thought to be the mechanism behind most of obesity-linked metabolic disorders [2].

One SR and one MA examined the effect of SSFR on multiple inflammatory markers such as TNF-α, C-reactive protein (CRP), and IL-6, and the findings are detailed in Table 2. In summary, the syntheses combined heterogeneous studies with different follow-up times. Conclusions varied between no change after SSFR or lower levels of IL-6 and TNF-α after surgery. However, the extent and time-trend were not reported, thus a dose response MA remains a needed future task.

**Table 2:** Syntheses that report changes in inflammatory markers after SSFR

	**Synthesis author and year**	**Synthesis type**	**Type of SSFR**	**Included studies**	**Follow up**	**Main finding**	**Remaining evidence gaps**
1	Sailon et al. 2017 [10]	SR	Liposuction	Four prospective studies (210 participants). The review examined the effect of large volume liposuction (more than 3.5 liters) on IL-6 and TNF-α	10 weeks–6 months	Two studies reported a statistically significant decrease in plasma IL-6 and TNF-α levels.	Neither a clear extent of change nor the clinical significance was reported.
2	Danilla et al. 2013 [39]	MA	Liposuction	Eight prospective studies (239 participants) examined the changes in CRP (4 studies), IL-6 (3 studies), and TNF-α (3 studies)	1–6 months	No association between the amount of aspirated fat and serum levels of CRP, IL-6, and TNF-α.	No clear report on the results, rather than just a general conclusion of no association.

SR: Systemic review; MA: Meta-analysis; CI: Confidence interval; CRP: C-reactive protein; IL-6: Interleukin 6; TNF-α: Tumor necrosis factor-alpha; SSFR: Surgical subcutaneous fat removal.

### Impact of SSFR on adipokine levels

Changes in the adipokines have been examined by only one SR and one MA, and both reported a reduction of leptin levels after SSFR. However, there was heterogeneity in the reported changes in other adipokines, such as adiponectin and resistin (Table 3).

**Table 3:** Syntheses that report changes in adipokines after SSFR

	**Synthesis author and year**	**Synthesis Type**	**Type of SSFR**	**Included studies**	**Follow up**	**Main finding**	**Remaining evidence gaps**
1	Sailon et al. 2017 [10]	SR	Liposuction	Five prospective studies (225 participants) examined the effect of large volume liposuction (>3.5 liters) on adipokines levels (namely leptin and adiponectin)	10 weeks–6 months	Leptin was examined by 4 studies, which all reported a statistically significant reduction. Adiponectin was assessed in all studies, two of which reported a significant increase.	Neither a clear extent of change, nor the clinical significance was reported. Other adipokines were not assessed.
2	Danilla et al. 2013 [39]	MA	Liposuction	Six quasi experiment studies (191 participants) examined the effect of SSFR on leptin levels	6 weeks–6 months	The MA showed a statistically significant reduction in leptin levels (Coefficient: 0.18). This reduction was proportional to the amount of aspirated fat, and patient BMI.	The study didn’t report the changes in other adipokines, nor the clinical significance of the reported changes.

SR: Systemic review; MA: Meta-analysis; SSFR: Surgical subcutaneous fat removal; BMI: Body mass index.

### Summary of findings

This umbrella review summarizes four attempts at evidence synthesis on the metabolic changes after surgical fat removal, with a total of 29 unique studies included and 759 total participants. There was a possible improvement in obesity-associated insulin resistance, however, there was a lack of clarity regarding the extent of the effect and clinical significance. Nevertheless, it seems likely that ASF removal is associated with improved insulin sensitivity. In terms of inflammation, one of the two syntheses reported that ASF removal results in a lower degree of IL-6 and TNF-α, and thus potentially a more favorable metabolic risk profile. These syntheses also reported a reduction of leptin levels after ASF removal through surgery. There was heterogeneity in the reported changes in other adipokines, such as adiponectin and resistin. Clearly, the data from the previous studies are not conclusive, nevertheless, it seems likely that SSFR is associated with improved insulin sensitivity and lower levels of inflammatory cytokines.

## Implications for future research

### The role of ASF vs AVF in human metabolism

The central obesity in the abdominal area represents one of the essential components of metabolic syndrome, along with insulin resistance, elevated serum triglyceride, blood pressure, and low high-density lipoproteins, and it is distributed between the ASF and AVF compartments [11] Although some studies have linked the metabolic risk of obesity mainly to the AVF tissue [16, 47], others have proposed that both AVF and ASF play a role in metabolic risk [10]. Generally, subcutaneous fat mass is more than twice the visceral fat mass, especially among females [48]. As a result, 85% of bloodstream free fatty acids are coming from the subcutaneous fat stores, which is a major contributor to systemic insulin resistance by inhibiting glucose uptake by skeletal muscles [49]. There is evidence from some studies among healthy men [50] and those with T2DM [51] that ASF may be more strongly correlated with insulin resistance than AVF. There has also been a report from a study of a healthy cohort of mixed genders that ASF correlates with insulin resistance independently of AVF, but not the other way around [52]. To sum this up, there is some evidence from the umbrella review as well as other studies suggesting that ASF may make an important contribution to obesity-related metabolic change, and this thus can be a mechanism through which SSFR can create a more favorable metabolic profile.

When studies have looked directly at the added impact of AVF on metabolism, by examining the effect of adding omentectomy to bariatric procedures, results were inconsistent. Some studies reported that it could result in better glucose homeostasis and lower inflammatory markers [53, 54]. Conversely, others reported a lack of clinical improvement in the metabolic profile [55–57]. Many open questions remain therefore about the role of AVF vs ASF and part of the problem lies in their study design, for example, the lack of clarity regarding patient selection, determining the type of surgery, the parameters that needed to be measured, and accounting for patient factors [58]. In addition, there were also technical limitations of older studies regarding advanced imaging technologies to measure visceral adipose tissue accurately. At a more fundamental level, improved knowledge of all aspects of adipose biology, including adipose tissue cellular heterogeneity [59, 60] as well as divergent responses to metabolic and endocrine stimuli that will be required to make significant advances and resolve the problem highlighted above [61]. In addition, a recent genome-wide association study also shows the contribution of genetics to visceral adiposity and its relation to ethnicities and gender in the context of metabolic disease. In particular, the study suggests that increased AVF is more harmful compared with ASF, but it is not clear why this should be the case [62].

### Adipokines

To determine why SSFR impacts adipokines levels, one needs to understand the roles of adipocyte-derived factors, as well as their effects on intermediary metabolism. Adipocyte-derived factors need to be understood in terms of source, relation to obesity, and main function. Tables 4 and 5 summarize the inflammatory and anti-inflammatory adipokines, the most well-known candidates are leptin and IL-6.

**Table 4:** Description of the potential inflammatory adipokines

	**Hormone**	**Source**	**Observed changes in obesity**	**Main function**
1	Leptin [89]	Mainly from adipocytes	Well-known marker of obesity	Satiety hormone that regulates body weight by suppressing the feeling of hunger, inhibits fat storage, and promotes fatty acid oxidization; also promotes inflammation
2	Resistin [90]	Adipocytes, monocytes, and macrophages	Increased in obesity, insulin resistance, and diabetic patients	Proinflammatory adipokine; thought to play a role in insulin resistance
3	Fatty acid binding protein-4 (FABP-4) [91]	Adipocytes and macrophages	Increased in obesity, insulin resistance, and diabetic patients	Plays a role insulin resistance and inflammation
4	Retinol binding protein (RBP-4) [92]	Adipocytes (especially visceral fat), macrophages, and liver	Increased in obesity, insulin resistance, and diabetic patients. Associated with hypertension, and dyslipidemia	Acts as a transporter for retinol and plays a role in insulin resistance development
5	Acylation stimulating protein (ASP) [93]	Adipocyte	Increased in obesity and dyslipidemia patients	Autocrine function that leads to increasing triglyceride synthesis
6	Lipocalin-2 (LCN2) [94]	Adipose tissue, liver, kidney, lung, macrophages, and neutrophils	Increased in obesity (especially in severely obese females)	Plays a role in inflammation and insulin resistance
7	Chemerin [95]	Adipose tissue, liver, as well as innate immune cells	Elevated with obesity and diabetic patients	Plays a role in insulin resistance, adipocyte metabolism, and diabetic induced cardiovascular disease
8	Visfatin [96]	Adipose tissue and neutrophils	Increased in obesity, and diabetic patients	Acts as a proinflammatory mediator
9	Vaspin [97]	Adipose tissue, liver, pancreas, stomach, muscles and skin	Increased in obesity, insulin resistance and diabetic patients	Acts as a member of the serine protease inhibitor family
10	Apelin [98]	Adipose tissue, hypothalamus, heart, and skeletal muscles	Increased in obesity, insulin resistance and diabetic patients	Plays a role in regulating glucose metabolism, by inducing glucose uptake
11	Gremlin-1 [99]	Preadipocytes	Increased in obesity	Acts as an inhibitor of bone morphogenetic protein (BMP), which is one of the transforming growth factor-beta family

### Leptin, ASF and insulin sensitivity

Leptin is a 167-residue peptide hormone encoded by the Ob gene, and it is secreted mainly by the adipocytes but also from the gastric epithelium and other tissues [63]. Since its identification in 1994 by positional cloning [64], leptin has gained much recognition as a crucial peripheral and central signaling molecule associated with energy balance. This, in turn, has contributed to changing the perception of the adipose tissue from being a form of passive energy depot (primarily in the form of energy-rich triglycerides (9 kilocalories per gram) to that of an active endocrine organ that actively modulates food intake and systemic energy metabolism.

Leptin levels are positively associated with BMI, HOMA-IR, and serum triglycerides and negatively with serum HDL in mostly normal weight health individuals suggesting that leptin increases with BMI as well as in those with insulin resistance [65]. The latter study suggests that leptin was coming mainly from ASF given correlation with hip and waist circumference but not with waist–hip ratio [65]. Under normal physiological conditions, bloodstream levels of leptin are proportional to fat mass for a given individual [66] suggesting that the increase in leptin is driven by fat mass and that both leptin and insulin resistance are consequences of an increase in fat mass. Nevertheless, basal plasma leptin concentrations are significantly lower in insulin-sensitive than in insulin-resistant men (1.90 ± 0.4 vs. 4.35 ± 1.21 ng/ml, *P* < 0.05) of identical body fat composition [67] suggesting either that excess leptin may also lead to increases in insulin resistance independent of adiposity or that leptin production increases in insulin resistant men in response to unknown feedback mechanisms in an effort to ameliorate the insulin resistance. The latter seems more plausible given that a direct action of leptin on its hypothalamic neuronal target is required to maintain normal glucose homeostasis data and insulin sensitivity [68, 69] and therefore the rising leptin level and insulin resistance in obesity lends plausibility to the conclusion that another fat derived molecule required for the leptin effect on glucose homeostasis may be downregulated in obesity for this paradoxical observation to hold. It remains to be determined if this molecule does indeed exist and what it could be.

**Table 5:** Description of the potential anti-inflammatory adipokines

	**Hormone**	**Source**	**Observed changes in obesity**	**Main function**
1	Adiponectin [100]	Adipose tissue and skeletal muscles	Lower levels in diabetic patients	Anti-obesity, anti-atherogenic, anti-inflammatory, and anti-diabetic effects
2	Omentin-1 [101]	Visceral adipose tissue	Lower levels in obese and diabetic patients	Anti-inflammatory, anti-obesity, anti-diabetic properties, and insulin sensitizing effects
3	Secreted frizzled related protein 5 (SSFRP5) [102, 103]	Adipose tissue	Lower levels in obese and diabetic patients	Anti-inflammatory and insulin sensitizing effects
4	Cardiotrophin-1 (CT-1) [103]	Adipose tissue, liver, kidney, muscle, heart, and lung, brain and testis	Controverial results regarding the changes in serum levels of obese patients	One of the IL-6 cytokine family, plays a role in glucose and lipid metabolism, has an insulin sensitizing potential effect

IL-6: Interleukin 6.

### Interleukin-6 (IL-6), ASF and inflammation

IL-6 is a 212-residue protein cytokine encoded by the IL-6 gene [70]. Since its identification in 1986 by molecular cloning of B-cell stimulatory factor-2 [71], IL-6 has been recognized as a cytokine with various biological activities implicated with a detrimental role in a wide range of inflammation-associated disease states, including susceptibility to diabetes mellitus [72]. IL-6 is synthesized by various cell types of which white adipocytes are responsible for one-third of basal serum levels in humans [73].

The IL-6 level is probably the single most important factor associated with the hepatic acute-phase response and this is a response to tissue damage or infection that initiates host defense mechanisms and whose goal is to eliminate the threat and facilitate tissue repair [74]. Obesity however is associated with chronic low-grade inflammation possibly from hypoxia in adipocytes, resulting in the release of IL-6 and activation of other factors that positively feedback and amplify IL-6 release [75]. This leads to the metabolic syndrome and similar to leptin, *in vitro* studies have shown that ASF produces more IL-6 than VSF [76] making the link between ASF and metabolic syndrome stronger than that for VSF [77].

### Leptin, IL-6 and the SSFR- bariatric surgery interaction

It is important to note that some SSFR patients tend to have had bariatric surgery, which is associated with enhanced postprandial gut hormone release, particularly GLP-1, a hormone interlinked with factors released from adipose tissue, e.g., leptin and IL-6 highlighted above. However, what remains unclear is whether or to what extent this crosstalk gets perturbed in patients undergoing SSFR and/or bariatric surgery. Furthermore, what are the long-term metabolic sequelae? Thus, a robust examination of the changes of IL-6 after the sudden removal of fat surgically by body contouring procedures might widen our understanding of the mechanisms behind these metabolic changes.

### Other considerations and future tasks

Apart from the potentially favorable effects of SSFR on metabolism and adipokines discussed above, many studies also support the effectiveness of bariatric surgery for treating obesity and weight-related disease [21, 78]. However, the question about the combined impacts of these surgical interventions has been relatively under-studied, and the results remain inconclusive. Future studies that can link the metabolic improvement after bariatric surgery and bariatric medications such as Semaglutide to the preferential loss of AVF or ASF will be of great benefit. Additionally, a dose response MAs is needed to examine the time trend of the metabolic changes after SSFR, which can answer important questions regarding the durability and extent of changes induced by these procedures over time.

When a negative energy balance is induced by interventions such as SSFR, resulting in a moderate initial reduction of 5% to 10% from baseline body weight, the physiological adaptations certainly favor weight regain; thus, most people recover weight post-SSFR or at the end of lifestyle interventions. With the common SSFR procedures, this loss is of abdominal fat that constitutes <15% of total adipose tissue [79], with the main component of the latter being ASF.

Given that fat distribution is one parameter that modifies the impact of obesity on health, knowledge about whether fat tissue removed through SSFR is replaced by new fat tissue and if this occurs in the same or at different anatomical sites is important since the latter may have worse effects. Previous studies reported that the fat could return to sites other than that from which fat has been removed, such as the breast, hip, and thigh regions [80, 81], but this is not always the case [82]. There is also the possibility that new fat may accumulate at sites where fat does not commonly accumulate (ectopic fat) and such ectopic adipose tissues may deposit in several organs/tissues (intramuscular/cardiac/hepatic) in the body with adverse consequences [83, 84]. However, recent studies of the heart [85, 86] have suggested that ectopic fat is protective against the risk of developing cardiovascular complications by increasing glycolysis, as a physiological healing response. In the context of SSFR, it is unclear to what extent the redistributed fat contributes to the ectopic fat accumulation in tissues, such as intramuscular, intrahepatic, and myocardial fat and if it has a protective or detrimental effect. Furthermore, it is unclear if and how or which specific factors drive the fat redistribution to ectopic regions in preference to the rest of the body spatiotemporally. Identifying such factors can be helpful surrogate biomarkers for predicting potential risk factors in epidemiological studies. However, it should be noted that rodent models of fat biology do not adequately represent what happens in humans, and higher mammals such as baboons may be a better model that closely resembles human adipocyte function [87].

Thus far, results from studies designed to identify the factors that address the regulation of energetics and body fat redistribution/ regeneration post-SSFR in rats, mice, or hamsters have limited contribution in closing the knowledge gap because of insufficient mechanistic data, inadequate sample size, or lack of proper statistical tests reported [88]. Therefore, future studies in appropriate animal models or human clinical trials should account for the biological consequences of ectopic fat redistribution following weight gain post-SSFR. However, there is a need to ascertain the beneficial or detrimental nature of fat redistribution at specific anatomical sites, in relation to its quantity, rate, and time of accumulation following weight gain post-SSFR.

## Conclusion

We conclude that there is a gap in terms of the probability of weight gain or accumulation of fat post-SSFR, but there is data that in the short term there might be a metabolic benefit of excess ASF removal. Longer-term data are needed to determine if this benefit is sustained in the longer term. Patients going for SSFR represent a unique population with a sudden removal of their ASF. However, the metabolic changes after these procedures are still unclear, and existing studies suggest a trend toward benefit rather than harm. There is thus no immediate harm from these procedures but there is a need for properly designed dose-response MAs as well as well-conducted prospective clinical studies to unravel these putative changes. In turn, this will help us not only to confirm the safety of these procedures but also to define if these procedures can be used for metabolic benefit and to broaden our knowledge about the mechanisms underpinning excess ASF and associated metabolic consequences.

## Supplemental Data
